# The complex HLA-E-nonapeptide in Behçet disease

**DOI:** 10.3389/fimmu.2023.1080047

**Published:** 2023-08-10

**Authors:** Ángel Luís Castaño-Núñez, Marco-Antonio Montes-Cano, José-Raúl García-Lozano, Norberto Ortego-Centeno, Francisco José García-Hernández, Gerard Espinosa, Genaro Graña-Gil, Juan Sánchez-Bursón, María Rosa Juliá, Roser Solans, Ricardo Blanco, Ana-Celia Barnosi-Marín, Ricardo Gómez de la Torre, Patricia Fanlo, Mónica Rodríguez-Carballeira, Luis Rodríguez-Rodríguez, Teresa Camps, Santos Castañeda, Juan-Jose Alegre-Sancho, Javier Martín, María Francisca González-Escribano

**Affiliations:** ^1^ Department of Immunology, Hospital Universitario Virgen del Rocío (IBiS, CSIC, US), Sevilla, Spain; ^2^ Department of Internal Medicine, Hospital Clínico San Cecilio, Granada, Spain; ^3^ Department of Internal Medicine, Hospital Universitario Virgen del Rocío, Sevilla, Spain; ^4^ Department Autoimmune Diseases, Hospital Universitari Clínic, Barcelona, Spain; ^5^ Department of Rheumatology, Complejo Hospitalario Universitario A Coruña, Coruña, Spain; ^6^ Department of Rheumatology, Hospital Universitario de Valme, Sevilla, Spain; ^7^ Department of Immunology, Hospital Universitari Son Espases, Palma de Mallorca, Spain; ^8^ Department of Internal Medicine, Autoimmune Systemic Diseases Unit, Hospital Vall d’Hebron, Universidad Autonoma de Barcelona, Barcelona, Spain; ^9^ Department of Rheumatology, Hospital Universitario Marqués de Valdecilla, Santander, Spain; ^10^ Department of Internal Medicine, Complejo Hospitalario Torrecárdenas, Almería, Spain; ^11^ Department of Internal Medicine, Hospital Universitario Central de Asturias, Oviedo, Spain; ^12^ Department of Internal Medicine, Hospital Virgen del Camino, Pamplona, Spain; ^13^ Deparment of Internal Medicine, Hospital Universitari Mútua Terrassa, Terrassa, Spain; ^14^ Department of Rheumatology, Hospital Clínico San Carlos, Madrid, Spain; ^15^ Department of Internal Medicine, Hospital Regional Universitario de Málaga, Málaga, Spain; ^16^ Department of Rheumatology, Hospital de la Princesa, IIS-Princesa, Madrid, Spain; ^17^ Department of Rheumatology, Hospital Universitario Doctor Peset, Valencia, Spain; ^18^ Instituto de Parasitología y Biomedicina “López-Neyra”, CSIC, PTS Granada, Granada, Spain

**Keywords:** Behçet disease, gene association, classical HLA Class I molecules, HLA-E, NK cells

## Abstract

**Introduction:**

The knowledge of the aetiology of Behçet disease (BD), an immune-mediated vasculitis, is limited. HLA-B, mainly HLA-B51, and HLA-A molecules are associated with disease, but the ultimate cause of this association remains obscure. There is evidence that NK cells participate in the etiopathology of BD. NK cells have activator and inhibitor surface receptors, like the KIR and the NKG2 families. Classical HLA-class I molecules (A, B and C) are keys in the activity control of the NK because they are KIR ligands. Most NKG2 receptors bind HLA-E, which presents only nonapeptides derived from the signal peptide of other class-I molecules.

**Objective:**

This study investigates the contribution of the pair HLA-E and ligand, nonapeptide derived from the 3-11 sequence of the signal peptides of class I classical molecules, to the susceptibility to BD.

**Methods:**

We analyzed the frequency of the HLA-derivated nonapeptide forms in 466 BD patients and 444 controls and an HLA-E functional dimorphism in a subgroup of patients and controls. Results: In B51 negative patients, the frequency of VMAPRTLLL was lower (70.4% versus 80.0% in controls; P=0.006, Pc=0.04, OR=0.60, 95%CI 0.41-0.86), and the frequency of VMAPRTLVL was higher (81.6% versus 71.4% in controls; P=0.004, Pc=0.03, OR=1.78, 95%CI 1.20-2.63). In homozygosity, VMAPRTLLL is protective, and VMAPRTLVL confers risk. The heterozygous condition is neutral. There were no significant differences in the distribution of the HLA-E dimorphism.

**Discussion:**

Our results explain the association of BD with diverse HLA-A molecules, reinforce the hypothesis of the involvement of the NK cells in the disease and do not suggest a significant contribution of the HLA-E polymorphism to disease susceptibility.

## Introduction

Behçet disease (BD) [Mendelian Inheritance in Man, MIM, 109650] is a complex and immune-mediated systemic syndrome featured by inflammatory lesions of various blood vessels throughout the body (most frequently small vessels). This chronic inflammation results in diverse clinical phenotypes: recurrent oral and genital ulceration, ocular involvement (mainly uveitis), skin lesions and others. Although BD aetiology remains obscure, some data suggest that infectious agents and environmental factors may trigger the disease in genetically predisposed individuals (1).

Likely in most immune-mediated diseases, the most substantial genetic contribution is located in the region of the Major Histocompatibility Complex (MHC, HLA in humans). Thus, one of the class I molecules, HLA-B51, associated with disease predisposition in different ethnic groups, is considered the most important genetic risk factor for this condition (2). Also, other HLA class I molecules are involved in susceptibility to BD (3–6). The ultimate cause of the association of HLA class I molecules and BD remain obscure. Diverse studies suggest that the natural killer (NK) cells participate in the etiopathology of the HLA class I-associated diseases (such as BD, spondyloarthritis and psoriasis) (7). NK cells are lymphocytes of the innate immune system with many activators and inhibitor receptors on their surface, like the Killer-cell Immunoglobulin-like Receptor (KIR) family. The KIR ligands are HLA class I molecules, and the interaction between the pair ligand-receptor is fundamental for the NK repertory selection and the regulation of cell activity. All the classical HLA-class I molecules (HLA-A, B and C) present peptides to the CD8+ T cells, but not all are KIR ligands. Several studies suggest that this role of the HLA-class I molecules, particularly HLA-B, could explain, at least partially, their association with diseases (4, 6, 8).

Besides KIR, other NK receptors control the activity of these cells. These type II- transmembrane proteins are a family of C-type lectin receptors, the NKG2 receptors, that form heterodimers with CD94 (CD94/NKG2). The human genes encoding the NKG2 receptors cluster in the natural killer complex (NKC) on chromosome 12. This protein family includes seven members: NKG2A, B, C, D, E, F and H, divided into inhibitory (NKG2A and B) and activating (NKG2 C, D, E, F and H) receptors according to their function. CD94 contains a short cytoplasmic domain, and it is responsible for signal transduction (9).

CD94/NKG2 binds HLA-E, a human nonclassical HLA class I molecule, except NKG2D, which binds other nonclassical HLA class I molecules, such as MIC-A and MIC-B. HLA-E is structurally similar to classical MHC class I molecules but present a very restricted set of peptides. In physiological conditions, HLA-E binds only a nonapeptide derived from the 3-11 sequence of the signal peptides of some class I molecules: (HLA-A, some B, C and G) but not from others (HLA-F and HLA-E (10). The load of the peptide to the HLA-E in the endoplasmic reticulum regulates the cell surface complex expression. NK cells can indirectly monitor the expression of classical MHC class I molecules through the CD94/NKG2 interaction with HLA-E. Only the HLA molecules with a leader peptide capable of upregulating HLA-E surface expression confer resistance to NK cell-mediated lysis for the inhibitory receptor CD94/NKG2A, predominantly expressed on the surface of NK cells and CD8+ T-lymphocyte. So, HLA-E molecules are involved in both innate and adaptive immunity. The HLA-E CD94/NKG2 system modulates either inhibition or activation of the NK cell-mediated cytotoxicity and cytokine production. In infection conditions, HLA-E can present microbial-derived peptides from viruses or bacteria, inducing T-cell responses (11). Contrasting to the classical HLA class I genes, HLA-E is minimally polymorphic. There are hundreds of alleles described (12), but the most common are two non-synonymous and functional HLA-E alleles worldwide distributed with similar frequency in the human population (approximately 50% each). These two alleles, HLA-E*01:01 and HLA-E*01:03, encode two proteins with a single amino acid substitution Arg107Gly (a2 domain of the heavy chain) (13). These two alleles differ in their quantitative cell surface expression, having HLA-E*01:03 higher concentrations on transfected cells when compared to HLA-E*01:01 (14). A single nucleotide polymorphism (SNP), rs1264457, permits classifying the HLA-E alleles into two groups, the most common of which are HLA-E 01:01 and 01:03.

This study investigates the contribution of the pair HLA-E and ligand, nonapeptide derived from the 3-11 sequence of the signal peptides of class I classical molecules, to the susceptibility to BD.

## Materials and methods

### Study population

The study included a total of 466 BD-unrelated patients (44.2% males) who fulfilled the 1990 International Study Group classification criteria for BD (15) and 444 unrelated healthy individuals (50% males) included in the control group. All the subjects were Spanish European recruited from 17 Spanish hospitals. The local ethical committees of the hospitals approved the study. All the study participants gave their written informed consent to participate. Clinical features of the patient group were the following: 100% had oral ulcers, 59.4% genital ulcers, 53.9% uveitis, 42% arthritis, 21% vascular, 18.2% neurological, 16.3% positive pathergy test and 15.4% gastrointestinal involvement.

### DNA extraction

Peripheral blood collected in EDTA tubes (healthy controls and a set of the patients) or saliva (part of the patients) served as starting material. Genomic DNA was extracted according to the manufacturer’s recommendations using the QIAamp DNA Mini Kit (Qiagen, Barcelona, Spain) and stored at −20°C until use. The purity of DNA was determined using a NanoDrop spectrophotometer (Thermo Fisher Scientific, Wilmington, DE, USA). Only DNA samples having a 260/280 absorbance ratio of 1.7-2.0 and a final concentration of 10-20 ng/μl were considered appropriate. We discarded 14 DNA samples from saliva that did not meet the quality criteria.

### Genotyping of HLA class I classical molecules

The present study includes data from the HLA class I (A, B and C) genotypes obtained in previous works of our group (6, 16). Briefly, we used a PCR-SSOP Luminex method (LABType SSO Class I A Locus Typing Test, LABType SSO Class I B Locus Typing Test and LABType SSO Class I C Locus Typing Test. One Lambda Inc., Canoga Park, CA) according to the manufacturer´s instructions to genotype. The DNA samples were PCR-amplified using group-specific primers (HLA-A, -B or -C). The biotinylated-PCR products were denatured and hybridized with specific probes bound to coloured-coded microspheres. Phycoerythrin conjugated to Streptoavidin was used to label and reveal reactions. The results were read in a flow analyzer, LABScanTM100, to quantify the fluorescence intensity on the microspheres. We used the HLA Fusion 2.0 software (One Lambda Inc) for the HLA-locus typing assignment. This method allows medium-resolution genotyping. **Table 1** displays the criteria used in sample classification according to the HLA-class I molecules nonamer leader 3-11 peptide sequence (17). The different nonamers are named as “N” plus number. The analyses included only samples with complete typing data in all three HLA class I loci (435 patients and 444 controls)

**Table 1:** Classification of the samples according to the different forms of the 3-11 nonapeptide sequence of the leader fragment of their HLA-class I classical molecules.

Group	Sequence	HLA-I allele groups
N1	VMAPRTLLL	A*01, *03, *11, *29, *30, *31, *32, *33, *36, *74; C*02, *15
N2	VMAPRTLVL	A*02, *23, *24, *25, *26, *34:02, *43, *66, *68, *69
N3	IMAPRTLVL	A*34:01
N4	VMPPRTLLL	A*80
N5	VMAPRTVLL	B*07, *08, *14, *38, *39, *42, *48, *67, *73, *81
N6	VTAPRTLLL	B*13, *18, *27, *37, *40:02, *40:06, *44, *47, *54, *55, *56, *59, *82, *83
N7	VTAPRTVLL	B*15, *35, *40:01, *41, *45, *46, *49, *50, *51, *52, *53, *57, *58, *78
N8	VMAPRTLIL	C*01, *03, *04, *05, *06, *08, *12, *14, *16, *17:02
N9	VMAPRALLL	C *07, *18
N10	VMAPQALLL	C*17 except C*17:02

Only sequences 3-11 present in common alleles in Caucasians are listed. Other sequence variants are only in uncommon alleles. For each variant, the allelic groups listed are those in which the most common alleles have this sequence.

The different nonamers are named as “N” plus number.

### Genotyping of the dimorphism of HLA- E

Genotyping of the rs1264457 (NM_005516.6: c.382A>G p.Arg128Gly correspond to mature protein codon 107) in a subgroup of 273 patients and 273 controls by using a TaqMan^®^ SNP Genotyping Assay (Applied Biosystems, Barcelona, Spain) in a LightCycler 480 (Roche, Barcelona, Spain). For comparing purposes, we classified the samples according to their genotyping result as HLA-E01:01, 01:01 (rs1264457AA), HLA-E01:01, 01:03 (rs1264457AG) and HLA-E*01:03. 01:03 (rs1264457GG).

### Statistical analysis

Phenotypic and genotypic frequencies were estimated by direct counting. In the univariate analyses, we used χ2. The P-values were corrected by Bonferroni adjustment (Pc), considering the number of tests in each case. The Pc-values<0.05 were considered statistically significant, and those with P-values<0.05 and Pc>0.05 were considered suggestive of association. EpiInfoTM software v 7.2.4.0 was used for the logistic regression analysis to calculate the odds ratios (ORs) and 95% confidence intervals (95% CI) (18).

## Results

The study population is at Hardy–Weinberg equilibrium (p>0.05) for the polymorphisms studied. **Supplementary Tables 1**–**3** display the phenotypical frequencies of the classical-HLA molecules in our population in the whole cohort and individuals B51 negative and B51 positive.

### Association of the different nonamer forms with BD

**Table 2** shows the frequencies of all the sequences 3-11 in the leader peptide of the HLA classical class I molecules in BD patients and healthy controls in our population. According to the univariate analysis, N2 and N7 frequencies are higher, whereas N5 is lower in patients than controls. After adjustment, only the N2 association remains significant. Then, as an additional way to reduce the problem of multiple testing, we constructed an unconditioned logistic regression model (**Supplementary Table 4**) with all the peptide forms (N1-N10) as independent dichotomous variables. Similarly, to the results with the Bonferroni adjustment, N2 resulted as the only associated peptide (P=0.01).

**Table 2:** Distribution of the nonapeptide-sequence types in BD patients and healthy controls.

Peptide	BD n= 435	%	Controls n= 444	%	P/Pc values	OR (95% CI)
N1	330	75.9	357	80.4		
N2	**360**	**82.8**	**328**	**73.9**	**0.001/0.01**	**1.70 (1.22-2.35)**
N3	2	0.5	0	0.0		
N4	1	0.2	2	0.5		
N5	**163**	**37.5**	**204**	**46.0**	**0.01/0.1**	**0.70 (0.53-0.92)**
N6	211	48.5	233	52.5		
N7	**348**	**80.0**	**319**	**71.9**	**0.006/0.06**	**1.57 (1.15-2.15)**
N8	380	87.4	388	87.4		
N9	159	36.6	169	38.1		
N10	7	1.6	11	2.5		

The table displays univariate analysis for each nonapeptide. Statistically significant comparisons are in bold. After adjustment by the number of tests, only N2 remains significantly associated.

Pc is the adjusted P-value.

### Nonamer forms in B51 positive and negative patients

Next, because HLA-B*51 is the most consistently disease-associated risk factor, we constructed different regression models by conditioning each peptide on B51. As a result, there were associations only for N1 and N2 (**Table 3**). Then, we divided the cohort into B51 positive and negative groups (**Table 4**). In the B51 negative group, N2 was a risk factor, whereas N1 conferred protection. Both associations were still significant after multiple test corrections. On the contrary, in the B51 positive, there were no significant differences in the distribution of the different peptides. The model of association of N1 and N2 is recessive, being N1N1 the protective and N2N2 the risk factors in the B51 negative individuals (**Table 5**).

**Table 3:** Results of the analyses conditioning the forms of the sequence of the nonapeptide 3-11 of the leader of the HLA class I classical molecules with frequency >5% on B*51.

Factor	P value	OR	95% CI
**B51**	**<10** ^-4^	**4.10**	2.98-5.66
**N1**	**0.02**	**0.66**	**0.47-0.93**
**B51**	**<10** ^-4^	**3.83**	**2.78-5.28**
**N2**	**0.02**	**1.51**	**1.07-2.12**
**B51**	**<10** ^-4^	**3.87**	**2.80-5.36**
N5	0.54		
**B51**	**<10** ^-4^	**4.00**	**2.90-5.53**
N6	0.68		
**B51**	**<10** ^-4^	**4.04**	**2.88-5.66**
N7	0.72		
**B51**	**<10^-4^**	**4.00**	**2.90-5.50**
N8	0.46		
**B51**	**<10^-4^**	**4.05**	**2.94-5.60**
N9	0.34		

Multivariate analysis of each nonapeptide conditioned on B51.

Statistically significant comparisons are in bold.

**Table 4:** Distribution of the variants of the sequence 3-11 of the leader of the HLA class I classical molecules with frequency >5% in the B51 positive and negative groups.

Peptide	B51 positive		B51 negative		P/Pc	OR (95% CI)
	BD (%) n= 185	Controls (%) n= 70	BD (%) n= 250	Controls (%) n= 374		
N1	154 (83.2)	58 (82.9)	**176 (70.4)**	**299 (80.0)**	**0.006/0.04**	**0.60 (0.41-0.86)**
N2	156 (84.3)	61 (87.1)	**204 (81.6)**	**267 (71.4)**	**0.004/0.03**	**1.78 (1.20-2.63)**
N5	45 (24.3)	17 (24.3)	118 (47.2)	187 (50.0)		
N6	63 (34.1)	30 (42.9)	148 (59.2)	203 (54.3)		
N7*	185 (100)	70 (100)	163 (65.2)	249 (66.6)		
N8	156 (84.3)	56 (80.0)	224 (89.6)	332 (88.8)		
N9	51 (27.6)	14 (20.0)	108 (43.2)	155 (41.4)		

Nonapeptide variants univariate analysis among B51-positive and B51-negative individuals.

* The N7 group includes B51.

Statistically significant comparisons are in bold.

Pc is the adjusted P-value.

**Table 5:** Genotypic frequencies of the different forms of the sequence 3-11 in the leader peptide of the HLA-A molecules in BD patients and healthy controls in our population.

HLA-A derivated-peptides	Whole cohort		P/Pc*	OR	95%CI
	BD (%) N=435	Controls (%) N=444			
**N1N1**	**74 (17.0)**	**116 (26.1)**	**0.001/0.006**	**0.6**	**0.4-0.8**
N1N2	206 (47.4)	221 (49.8)			
N1N3	1 (0.2)	0 (0.0)			
**N2N2**	**152 (34.9)**	**105 (23.7)**	**0.0002/0.001**	**1.7**	**1.3-2.3**
N2N3	1 (0.2)	0 (0.0)			
N2N4	1 (0.2)	2 (0.5)			
	B51 negative				
	BD (%) N=250	Controls (%) N=374			
**N1N1**	**45 (18.0)**	**107 (28.6)**	**0.003/0.02**	**0.6**	**0.4-0.8**
N1N2	117 (46.8)	182 (48.7)			
N1N3	1 (0.4)	0 (0.0)			
**N2N2**	**85 (34.0)**	**83 (22.2)**	**0.001/0.006**	**1.8**	**1.2-2.6**
N2N3	1 (0.4)	0 (0.0)			
N2N4	1 (0.4)	2 (0.5)			
	B51 positive				
	BD (%) N=185	Controls (%) N=70			
N1N1	29 (15.7)	9 (12.7)			
N1N2	89 (48.1)	39 (55.7)			
N1N3	0 (0.0)	0 (0.0)			
N2N2	67 (36.2)	22 (31.4)			
N2N3	0 (0.0)	0 (0.0)			
N2N4	0 (0.0)	0 (0.0)			

Univariate analysis of the HLA-A nonapeptide genotypes in the whole group and B51 negative and positive individuals.

Statistically significant comparisons are in bold.

* P/Pc for the recessive models (AA versus Aa+aa). For the dominant models (AA+Aa versus aa) P>0.05. Pc is the adjusted P-value.

Since different nonamer forms include BD-associated HLA molecules, we realized several conditional analyses. So, N1 contains A*03, a protective factor for susceptibility to BD, in various studies in several populations. For this reason, we conditioned N1 on HLA-A*03 in the B*51 positive and negative groups. In the case of B*51 positive, A*03 remains significant, and N1 lost statistical significance. Otherwise, in the B51 negative group, N1 remains significant while A*03 lost the association (**Table 6**). Another way, N2 includes A*02, A24 and A*26, which are HLA molecules associated with the disease in various studies. Conditioning N2 on these factors, in the group of B51 carriers, N2 lacks association in all the cases, and none of the HLA-A molecules mentioned above had an association. Nevertheless, among B51-negative, N2 remains associated with conditioning on A24 and A26. For A2 and N2, both factors lack association (**Table 6**).

**Table 6:** Results of the analyses conditioning N1 on A*03 and N2 on A*02, A*24 and A*26 in the B*51 positive and negative groups.

B51-positive individuals			
Factor	p	OR	95% CI
N1	0.65		
**A3**	**0.02**	**0.35**	**0.15-0.83**
N2	0.73		
A2	0.83		
N2	0.46		
A24	0.32		
N2	0.59		
A26	0.84		
B51-negative individuals			
Factor	p	OR	95% CI
**N1**	**0.02**	**0.64**	**0.44-0.94**
A3	0.15		
N2	0.09		
A2	0.11		
**N2**	**0.007**	**1.75**	**1.17-2.62**
A24	0.77		
**N2**	**0.006**	**1.75**	**1.18-2.60**
A26	0.66		

Multivariate analysis by conditioning N1 and N2 nonapeptides on the related to disease susceptibility HLA-A classical molecules included in the corresponding group (See **Table 1**).

Statistically significant comparisons are in bold.

### The functional 2-position of the nonapeptide and BD

The nonapeptide position 2, which is polymorphic only in HLA-B molecules, is functionally relevant because the 2Met-HLA-B nonapeptides bind more efficiently to HLA-E when compared to the Thr variant. The distribution of this dimorphism in our cohort has significant differences between patients and controls in the univariate analysis, with risk association of 2Thr-HLA-B in a recessive model that became non-significant by correcting on B51 and also stratifying by B51 positive and negative (**Supplementary Table 5**).

### HLA-E polymorphism and BD

Regarding polymorphism of HLA-E, although the patient group tends to have a higher frequency of the genotype 01:01, 01:01, there were no significant differences in the distribution of genotypes, phenotypes (dominant and recessive models) and alleles (**Table 7**). Neither when categorizing individuals in B51 positive and negative (**Supplementary Table 6**) nor stratifying by the different types of 3-11 leader sequence peptides (**Supplementary Table 7**).

**Table 7:** HLA-E genotypes, phenotypes and alleles in BD patients and controls.

Genotypes	BD (%) n= 273	Controls (%) n= 273
01:01,01:01	83 (30.4)	72 (26.4)
01:01,01:03	129 (47.2)	139 (50.9)
01:03,01:03	61 (22.3)	62 (22.7)
Dominant Model		
01:01	212 (77.7)	211 (77.3)
01:03	190 (69.6)	201 (73.6)
Recessive Model		
01:01	83 (30.4)	72 (26.4)
01:03	61 (22.3)	62 (22.7)
Allelic Model	2n= 546	2n= 546
01:01	295 (0.54)	283 (0.52)
01:03	251 (0.46)	263 (0.48)

HLA-E genotypes univariate analysis with different inheritance models.

Dominant model AA+Aa versus aa; Recessive model AA versus Aa+aa

All the P values are >0.05.

## Discussion

This study investigates the contribution of the pair HLA-E and ligand, nonapeptide derived from the 3-11 sequence of the signal peptides of class I classical molecules, to the susceptibility to BD. The main finding is the association between some forms of nonapeptide and the disease in B51-negative patients. In this group of patients, the form VMAPRTLLL (N1) confers protection, while the form VMAPRTLVL (N2) confers risk to the susceptibility to the disease in a recessive model where the genotypes are: N1N1 protective, N1N2 neutral and N2N2 risk.

In the univariate analysis of the whole cohort, there were three forms of the nonapeptide related to BD (N2, N5 and N7). However, data from logistic regression analysis, including all sequences in the model, suggest an independent association only for N2. It is necessary to consider diverse confounding factors in the HLA region analysis. For example, N5 includes HLA-B molecules that do not associate with BD, whereas N7 contains HLA-B51, the main BD risk factor. In the multivariate analysis and when corrected on HLA-51, only N2 remains an independently associated factor. Analysis of the whole cohort did not detect any association of N1 with the disease. When stratifying the cohort in B51 positive and negative individuals, the N2 association remains only among B51-negative. The molecules HLA-A2, A24 and A26, included in N2, have been associated with the disease in different studies, although with controversial results even within the same population (3, 4, 6, 16, 19–21). The association of the 3-11 VMAPRTLVL sequence (N2) of the leader peptide in the B51 negative group could explain the discrepancies regarding the association of BD with diverse HLA-A molecules because the specific molecule associated would depend on the cohort characteristics. The interdependence of the A2 and N2 associations may be due to the high frequency of A2. The relationship of N1 with BD became apparent only when splitting according to the presence of B51. The N1 group includes HLA-A3, a protective factor associated with BD in many studies (4, 6, 16, 19–22). According to our results, among B51-positive individuals, the HLA-A3 molecule explains protection against the disease. Nevertheless, among the B51-negative, the form VMAPRTLLL (N1) in the 3-11 sequence of the leader peptide explains the association better. So HLA-A3 is protective in both groups, in B51 carriers by itself, and in B51 non-carriers as a molecule of the N1 group.

The primary function of the complex HLA-E/nonapeptide is to control NK activity by binding the complex to receptors CD94/NKG2 present on the surface of these cells and specific subpopulations of CD8 T cells. This interaction regulates cytotoxic activity by balancing inhibition (CD94/NKG2A) and activation (CD94/NKG2C). The HLA-derivates nonapeptides modulate this function in two aspects: First, the nonapeptide uploaded directs the trafficking to the membrane, and second, the peptides are essential factors for the HLA-E to receptors binding (10, 23, 24). Concerning the first point, HLA-E molecules will only be exported to the cell surface whenever they have a charged peptide. Therefore, the different affinities of the diverse forms of the nonapeptides affect the HLA-E expression. The nonapeptide primary amino acids involved in HLA-E binding are those at positions 2 and 9 (10, 25). The residue 9, Leu, is invariant in all the HLA-class I classical molecules, but the amino acid at 2-position can be Thr or Met (17). 2Met-HLA leader peptides bind more efficiently to HLA-E when compared to the Thr variant (14, 25, 26). The HLA-A and C derivate nonapeptides have 2Met, but the HLA-B can have 2Met (N5 in this study) and 2Thr (N6 and N7). Accordingly, individuals with 2Met-HLA-B nonapeptides have a higher surface expression of HLA-E with consequently more potent NKG2A-mediated NK-cell inhibitory properties (10, 14, 25, 26). Our results do not support an association of the HLA-B molecules based on the HLA-E expression modulation because the differences detected in the 2Met/Thr dimorphism distribution in patients and controls depend on B51. Regarding the different forms of the nonapeptide 2Met, according to our results, the secondary anchor residues are not associated: N1, N2, N3 and N8 have the same sequence in these positions (3Ala 6Thr 7 Leu), N3, which is rare, and N8, more common, were not associated, and associations of N1 and N2 were contrary, protective and risk, respectively. Concerning the second point, the interaction of the complex HLA-E/peptide with the receptor NKG2, the side chains fully exposed to receptors are residues 4, 5, and 8, whereas residues 1 and 6 are much less exposed. Mutational analyses suggest that the conserved Arg5 and the variable hydrophobic residue at position 8 are the primary contacts for CD94/NKG2A. The four C-terminal nonapeptide last-positions interact with CD94 and modify between 8 and 29 times the affinity for NKG2, attributing the maximum effect to position 8 (27–30). Models of the complex interactions suggest that even conserved substitutions at the nonapeptide 8-position significantly affect the affinity of the ligand for its receptors (28). HLA-B-derived nonapeptides have 8Leu, whereas the HLA-C derived have Ile (N8) and Leu (N9 and N10), but none associated with susceptibility in our study. Also, there are two HLA-A-derived nonapeptide groups, 8Leu (N1, protection) and 8Val (N2, risk). Like N1, the nonapeptides N9 and N10 have 2Met and 8Leu. The lack of association observed in these cases may be related to poor expression of the HLA-C molecules at the cell surface compared with HLA-A and HLA-B molecules (31) and specifically of the encoding by C*07 alleles, the most frequent of the included in N9 and N10 (32). Therefore, our results support that the association with BD described in the HLA-A region in several studies is related to the controls of the NK activity through the interaction of the ligand HLA-E-peptide and NKG2 receptor but only in patients B51 negative.

There is evidence of two predominant HLA haplotype forms, one preferentially supplying CD94/NKG2A ligands and the other providing KIR ones (33, 34). Many HLA-B molecules with 2Thr derivate nonapeptides have the Bw4 epitope (spans the residues 77-83), so they are KIR3DL1 ligands. Conversely, most of the 2Met HLA-B molecules are not KIR ligands. Accordingly, functional studies suggest that Met/Met or Met/Thr individuals have NK cells better educated, phenotypically more diverse and functionally more potent than those from individuals Thr/Thr (34). HLA-B51 is a 2Thr HLA-B molecule with the Bw4 epitope, like many others in our N6 and N7 groups. Our results do not support a model of HLA-B association related to the NK control activity through CD94/NKG2A. Previous studies suggested different hypotheses involving the interaction of HLA-Bw4-KIR with susceptibility to BD (4, 6). The HLA-C molecules all supply CD94/NKG2A ligands and are KIR ligands: C1 (those with Asn80) or C2 (with Lys80) are ligands of KIR2DL2 and KIR2DL1, respectively. Our results discard the association of these HLA molecules related to their function of CD94/NKG2A ligands. All the HLA-A molecules are 2Met, and the group N2 includes several molecules with the Bw4 epitope (A23, 24, 25 and 26). N1 contains only one Bw4 molecule (A32) and another two HLA molecules, A3 and A11, KIR3DL2 ligands. Therefore, all the HLA-A molecules supply CD94/NKG2A ligands, but only some are KIR ligands. Our results support the association of the HLA-A region with BD in B51-negative individuals based on its function as CD94/NKG2A-ligand. These results also suggest a different mechanism operating in individuals B51 positive and negative, with more weight of the interactions HLA-KIR among the positive and CD94/HLAE-NKG2A among the negative. Notable, HLA-A3 itself is protective in B51 individuals, whereas, in the B51 negative group, the association remain, although as a member of N1. These results may support a different base of the protective role of this molecule, based on the selection of licensed KIR3DL2 NK cells in B51 positive and in its function as CD94/NKG2A-ligand in B51 negative individuals. Our results, needing confirmation in other cohorts, generate hypotheses about the role of the HLA-E nonapeptide repertoire in BD. These hypotheses need testing in functional studies with NK cells, and they are of interest in other diseases associated with HLA class I.

There are many HLA-E alleles (specifically 311 encoding 121 different proteins) (12), but most are considered rare variants or non-functional proteins, with groups 01:01 and 01:03 representing almost 100% of alleles (35). Even though there is a trend to a slightly higher frequency of HLA-E*01:01 among patients, our results, with similar frequencies in patients and controls, do not suggest any association of these two HLA-E alleles with BD in any inheritance models. The association between this HLA-E dimorphism and different immune-mediated diseases is unclear (36–40). Our results disagree with a previously published study in a BD Korean cohort. The study in the Korean cohort, similar in size to the one included in the present study, suggests a protective effect of the HLA-E*01:01 in homozygosis (41). Although we did not detect significant differences in our cohort, HLA-E*01:01, 01:01 frequency tends to be higher in patients, suggesting opposite effects.

In conclusion, our results, which require confirmation in functional studies, support the association of the nonapeptide sequence of the HLA-A molecules with the susceptibility to BD in B51-negative individuals. These results reinforce the hypothesis of the involvement of the NK cells´ education and activity in the pathogenesis of the disease. The association is compatible with a recessive model in which the form VMAPRTLLL confers protection and the VMAPRTLVL risk susceptibility to the disease. These results suggest a different weight of the interactions between HLA and KIR and the complex HLA-E-peptide with NKG2/CD94 in B51 positive and negative individuals. This study does not suggest a significant contribution of the HLA-E polymorphism to disease susceptibility.

## Data availability statement

The datasets presented in this study can be found in the online repository NCBI https://www.ncbi.nlm.nih.gov/bioproject?term=%20PRJNA1001001.

## Ethics statement

The studies involving human participants were reviewed and approved by CEI de los hospitales universitarios Vírgen Macarena-Virgen del Rocío. The patients/participants provided their written informed consent to participate in this study.

## Author contributions

Experimental design: MFG-E. Experimental procedures performance: AC-N. Research support: J-RG-L, M-AM-C. Data analysis: AC-N, J-RG-L, M-AMC-, MFG-E. Preparation of the manuscript: AC-N, J-RG-L, M-AM-C, MFG-E. Samples and clinical data providers: NO-C, FJG-H, GE, GG-G, JS-B, MR, RS, RB, A-CB-M, RG, PF, MR-C, LR-R, TC, SC, J-JA-S and JM. All authors contributed to the article and approved the submitted version.
