# Supplementary material for: The complex HLA-E-nonapeptide in Behçet disease
Source: Front Immunol. 2023 Aug 10;14:1080047. doi: 10.3389/fimmu.2023.1080047 (PMC10449640; doi:10.3389/fimmu.2023.1080047)
Supplement: Supplementary file 1 [file Table_1.doc]

**Supplementary Table 1.** Phenotypical frequencies of the classical HLA class I molecules in our whole cohort.

|  | BD (%)  N=435 | Controls (%)  N=444 | P | OR | 95% CI |
| --- | --- | --- | --- | --- | --- |
| A*01 | 78 (17.9) | 84 (18.9) |  |  |  |
| A*02 | 247 (56.8) | 196 (44.1) | 0.0002 | 1.7 | 1.3-2.2 |
| A*03 | 47 (10.8) | 87 (19.6) | 0.0003 | 0.5 | 0.3-0.7 |
| A*11 | 45 (10.3) | 61 (13.7) |  |  |  |
| A*23 | 33 (7.6) | 31 (7.0) |  |  |  |
| A*24 | 87 (20.0) | 72 (16.2) |  |  |  |
| A*25 | 7 (1.6) | 11 (2.5) |  |  |  |
| A*26 | 38 (8.7) | 34 (7.7) |  |  |  |
| A*29 | 57 (13.1) | 69 (15.5) |  |  |  |
| A*30 | 35 (8.1) | 46 (10.4) |  |  |  |
| A*31 | 31 (7.1) | 23 (5.2) |  |  |  |
| A*32 | 30 (6.9) | 38 (8.6) |  |  |  |
| A*33 | 17 (3.9) | 30 (6.7) |  |  |  |
| A*34 | 2 (0.5) | 0 (0.0) |  |  |  |
| A*36 | 1 (0.2) | 0 (0.0) |  |  |  |
| A*66 | 9 (2.1) | 7 (1.6) |  |  |  |
| A*68 | 28 (6.4) | 50 (11.3) |  |  |  |
| A*69 | 1 (0.2) | 4 (0.9) |  |  |  |
| A*80 | 1 (0.2) | 2 (0.5) |  |  |  |
| B*07 | 54 (12.4) | 59 (13.3) |  |  |  |
| B*08 | 37 (8.5) | 40 (9.0) |  |  |  |
| B*13 | 11 (2.5) | 10 (2.3) |  |  |  |
| B*14 | 53 (12.2) | 71 (16.0) |  |  |  |
| B*15 | 59 (13.6) | 30 (6.8) | 0.0008 | 2.2 | 1.4-3.5 |
| B*18 | 47 (10.8) | 70 (15.8) | 0.03 | 0.7 | 0.4-1.0 |
| B*27 | 29 (6.7) | 26 (5.9) |  |  |  |
| B*35 | 51 (11.7) | 97 (21.8) | 0.00006 | 0.5 | 0.3-0.7 |
| B*37 | 4 (0.9) | 5 (1.1) |  |  |  |
| B*38 | 18 (4.1) | 31 (7.0) |  |  |  |
| B*39 | 10 (2.3) | 16 (3.6) |  |  |  |
| B*40:01 | 20 (4.6) | 27 (6.1) |  |  |  |
| B*40:02 | 0 | 2 (0.4) |  |  |  |
| B*41 | 8 (1.8) | 12 (2.7) |  |  |  |
| B*42 | 1 (0.2) | 1 (0.2) |  |  |  |
| B*44 | 112 (25.7) | 112 (25.2) |  |  |  |
| B*45 | 13 (3.0) | 21 (4.7) |  |  |  |
| B*46 | 1 (0.2) | 0 (0.0) |  |  |  |
| B*47 | 1 (0.2) | 1 (0.2) |  |  |  |
| B*48 | 0 (0.0) | 2 (0.5) |  |  |  |
| B*49 | 27 (6.2) | 41 (9.2) |  |  |  |
| B*50 | 18 (4.1) | 32 (7.2) | 0.05 | 0.6 | 0.3-1.0 |
| B*51 | 185 (42.5) | 69 (15.5) | <10^-8^ | 4.0 | 2.9-5.6 |
| B*52 | 9 (2.1) | 12 (2.7) |  |  |  |
| B*53 | 16 (3.7) | 16 (3.6) |  |  |  |
| B*54 | 0 (0.0) | 1 (0.2) |  |  |  |
| B*55 | 7 (1.6) | 6 (1.4) |  |  |  |
| B*56 | 4 (0.9) | 5 (1.1) |  |  |  |
| B*57 | 40 (9.2) | 20 (4.5) | 0.005 | 2.2 | 1.2-3.8 |
| B*58 | 3 (0.7) | 15 (3.4) | 0.005 | 0.2 | 0.1-0.6 |
| C*01 | 25 (6.0) | 23 (5.2) |  |  |  |
| C*02 | 58 (13.3) | 43 (9.7) |  |  |  |
| C*03 | 47 (10.8) | 40 (9.0) |  |  |  |
| C*04 | 84 (19.3) | 120 (27.0) | 0.007 | 0.6 | 0.5-0.9 |
| C*05 | 71 (16.3) | 79 (17.8) |  |  |  |
| C*06 | 64 (14.7) | 66 (14.9) |  |  |  |
| C*07 | 158 (36.3) | 168 (37.8) |  |  |  |
| C*08 | 58 (13.3) | 74 (16.7) |  |  |  |
| C*12 | 42 (9.7) | 73 (16.4) | 0.002 | 0.6 | 0.4-0.8 |
| C*14 | 33 (7.6) | 13 (2.9) | 0.002 | 2.7 | 1.4-5.4 |
| C*15 | 79 (18.2) | 35 (7.9) | 0.00001 | 2.6 | 1.7-4.0 |
| C*16 | 98 (22.5) | 77 (17.3) | 0.05 | 1.4 | 1.0-1.9 |
| C*17 | 7 (1.6) | 11 (2.5) |  |  |  |
| C*18 | 1 (0.2) | 1 (0.2) |  |  |  |

The Table displays the univariate analysis for each HLA molecule. P-values are uncorrected.

**Supplementary Table 2.** Phenotypical frequencies of the classical HLA class I molecules in B51- negative individuals.

|  | BD (%)  N=250 | Controls (%)  N=374 | P | OR | 95% CI |
| --- | --- | --- | --- | --- | --- |
| A*01 | 48 (19.2) | 75 (20.1) |  |  |  |
| A*02 | 129 (51.6) | 149 (39.8) | 0.004 | 1.6 | 1.2-2.2 |
| A*03 | 34 (13.6) | 75 (20.1) | 0.04 | 0.6 | 0.4-1.0 |
| A*11 | 21 (8.4) | 53 (14.2) | 0.03 | 0.6 | 0.3-0.9 |
| A*23 | 20 (8.0) | 29 (7.8) |  |  |  |
| A*24 | 49 (19.6) | 61 (16.3) |  |  |  |
| A*25 | 5 (2.0) | 9 (2.4) |  |  |  |
| A*26 | 24 (9.6) | 28 (7.5) |  |  |  |
| A*29 | 31 (12.4) | 62 (16.6) |  |  |  |
| A*30 | 22 (8.8) | 41 (11.0) |  |  |  |
| A*31 | 14 (5.6) | 14 (3.7) |  |  |  |
| A*32 | 13 (5.2) | 35 (9.4) |  |  |  |
| A*33 | 13 (5.2) | 27 (7.2) |  |  |  |
| A*34 | 2 (0.8) | 0 (0.0) |  |  |  |
| A*36 | 1 (0.4) | 0 (0.0) |  |  |  |
| A*66 | 8 (3.2) | 5 (1.3) |  |  |  |
| A*68 | 24 (9.6) | 41 (11.0) |  |  |  |
| A*69 | 1 (0.4) | 4 (1.2) |  |  |  |
| A*80 | 1 (0.4) | 2 (0.5) |  |  |  |
| B*07 | 37 (14.8) | 57 (15.2) |  |  |  |
| B*08 | 25 (10.0) | 35 (9.4) |  |  |  |
| B*13 | 8 (3.2) | 9 (2.4) |  |  |  |
| B*14 | 40 (16.0) | 66 (17.6) |  |  |  |
| B*15 | 46 (18.4) | 28 (7.5) | 0.00003 | 2.8 | 1.7-4.6 |
| B*18 | 31 (12.4) | 62 (16.6) |  |  |  |
| B*27 | 24 (9.6) | 23 (6.2) |  |  |  |
| B*35 | 35 (14.0) | 88 (23.5) | 0.003 | 0.5 | 0.3-0.8 |
| B*37 | 3 (1.2) | 4 (1.1) |  |  |  |
| B*38 | 16 (6.4) | 29 (7.8) |  |  |  |
| B*39 | 9 (3.6) | 14 (3.7) |  |  |  |
| B*40:01 | 15 (6.0) | 25 (6.7) |  |  |  |
| B*40:02 | 0 (0.0) | 2 (0.5) |  |  |  |
| B*41 | 8 (3.2) | 10 (2.7) |  |  |  |
| B*42 | 1 (0.4) | 1 (0.3) |  |  |  |
| B*44 | 81 (32.4) | 99 (26.5) |  |  |  |
| B*45 | 9 (3.6) | 19 (5.1) |  |  |  |
| B*46 | 1 (0.4) | 0 (0.0) |  |  |  |
| B*47 | 1 (0.4) | 1 (0.3) |  |  |  |
| B*48 | 0 (0.0) | 2 (0.5) |  |  |  |
| B*49 | 20 (8.0) | 41 (11.0) |  |  |  |
| B*50 | 10 (4.0) | 29 (7.8) |  |  |  |
| B*52 | 7 (2.8) | 11 (2.9) |  |  |  |
| B*53 | 14 (5.6) | 16 (4.3) |  |  |  |
| B*54 | 0 (0.0) | 1 (0.3) |  |  |  |
| B*55 | 5 (2.0) | 4 (1.1) |  |  |  |
| B*56 | 4 (1.6) | 5 (1.3) |  |  |  |
| B*57 | 29 (11.6) | 20 (5.4) |  |  |  |
| B*58 | 2 (0.8) | 14 (3.7) |  |  |  |
| C*01 | 16 (6.4) | 17 (4.5) |  |  |  |
| C*02 | 28 (11.2) | 28 (7.5) |  |  |  |
| C*03 | 31 (12.2) | 36 (9.6) |  |  |  |
| C*04 | 56 (22.4) | 107 (28.6) |  |  |  |
| C*05 | 49 (19.6) | 69 (18.5) |  |  |  |
| C*06 | 46 (18.4) | 60 (16.0) |  |  |  |
| C*07 | 107 (42.8) | 154 (41.2) |  |  |  |
| C*08 | 42 (16.8) | 69 (18.5) |  |  |  |
| C*12 | 32 (12.8) | 66 (17.6) |  |  |  |
| C*14 | 1 (0.4) | 2 (0.5) |  |  |  |
| C*15 | 6 (2.4) | 15 (4.0) |  |  |  |
| C*16 | 44 (17.6) | 56 (15.0) |  |  |  |
| C*17 | 7 (2.8) | 9 (2.4) |  |  |  |
| C*18 | 1 (0.4) | 1 (0.3) |  |  |  |

The Table displays the univariate analysis for each HLA molecule. P-values are uncorrected.

**Supplementary Table 3.** Phenotypical frequencies of the classical HLA class I molecules in B51- positive individuals.

|  | BD (%)  N=185 | Controls (%)  N=70 | P | OR | 95% CI |
| --- | --- | --- | --- | --- | --- |
| A*01 | 30 (16.2) | 9 (12.9) |  |  |  |
| A*02 | 118 (63.8) | 47 (67.1) |  |  |  |
| A*03 | 13 (7.0) | 12 (17.1) | 0.02 | 0.4 | 0.2-0.9 |
| A*11 | 24 (13.0) | 8 (11.4) |  |  |  |
| A*23 | 13 (7.0) | 2 (2.9) |  |  |  |
| A*24 | 38 (20.5) | 11 (15.7) |  |  |  |
| A*25 | 2 (1.1) | 2 (2.9) |  |  |  |
| A*26 | 14 (7.6) | 6 (8.6) |  |  |  |
| A*29 | 26 (14.1) | 7 (10.0) |  |  |  |
| A*30 | 13 (7.0) | 5 (7.1) |  |  |  |
| A*31 | 17 (9.2) | 9 (12.9) |  |  |  |
| A*32 | 17 (9.2) | 3 (4.3) |  |  |  |
| A*33 | 4 (2.2) | 3 (4.3) |  |  |  |
| A*34 | 0 (0.0) | 0 (0.0) |  |  |  |
| A*36 | 0 (0.0) | 0 (0.0) |  |  |  |
| A*66 | 1 (0.5) | 2 (2.9) |  |  |  |
| A*68 | 4 (2.2) | 9 (12.9) |  |  |  |
| A*69 | 0 (0.0) | 0 (0.0) |  |  |  |
| A*80 | 0 (0.0) | 0 (0.0) |  |  |  |
| B*07 | 17 (9.2) | 2 (2.9) |  |  |  |
| B*08 | 12 (6.5) | 5 (7.1) |  |  |  |
| B*13 | 3 (1.6) | 1 (1.4) |  |  |  |
| B*14 | 13 (7.0) | 5 (7.1) |  |  |  |
| B*15 | 13 (7.0) | 2 (2.9) |  |  |  |
| B*18 | 16 (8.7) | 8 (11.4) |  |  |  |
| B*27 | 5 (2.7) | 3 (4.3) |  |  |  |
| B*35 | 16 (8.7) | 9 (12.9) |  |  |  |
| B*37 | 1 (0.5) | 1 (1.4) |  |  |  |
| B*38 | 2 (1.1) | 3 (4.3) |  |  |  |
| B*39 | 1 (0.5) | 2 (2.9) |  |  |  |
| B*40:01 | 5 (2.7) | 2 (2.9) |  |  |  |
| B*40:02 | 0 (0.0) | 0 (0.0) |  |  |  |
| B*41 | 0 (0.0) | 2 (2.9) |  |  |  |
| B*42 | 0 (0.0) | 0 (0.0) |  |  |  |
| B*44 | 31 (16.8) | 13 (18.6) |  |  |  |
| B*45 | 4 (2.2) | 2 (2.9) |  |  |  |
| B*46 | 0 (0.0) | 0 (0.0) |  |  |  |
| B*47 | 0 (0.0) | 0 (0.0) |  |  |  |
| B*48 | 0 (0.0) | 0 (0.0) |  |  |  |
| B*49 | 7 (3.8) | 0 (0.0) |  |  |  |
| B*50 | 8 (4.3) | 3 (4.3) |  |  |  |
| B*52 | 2 (1.1) | 1 (1.4) |  |  |  |
| B*53 | 2(1.1) | 0 (0.0) |  |  |  |
| B*54 | 0 (0.0) | 0 (0.0) |  |  |  |
| B*55 | 2 (1.1) | 2 (2.9) |  |  |  |
| B*56 | 0 (0.0) | 0 (0.0) |  |  |  |
| B*57 | 11 (5.9( | 0 (0.0) |  |  |  |
| B*58 | 1 (0.5) | 1 (1.4) |  |  |  |
| C*01 | 9 (4.9) | 6 (8.6) |  |  |  |
| C*02 | 30 (16.2) | 15 (21.4) |  |  |  |
| C*03 | 16 (8.6) | 4 (5.7) |  |  |  |
| C*04 | 28 (15.1) | 13 (18.6) |  |  |  |
| C*05 | 22 (11.9) | 10 (14.3) |  |  |  |
| C*06 | 18 (9.7) | 6 (8.6) |  |  |  |
| C*07 | 51 (27.6) | 14 (20.0) |  |  |  |
| C*08 | 16 (8.6) | 5 (7.1) |  |  |  |
| C*12 | 10 (5.4) | 7 (10.0) |  |  |  |
| C*14 | 32 (17.3) | 11 (15.7) |  |  |  |
| C*15 | 73 (39.5) | 20 (28.6) |  |  |  |
| C*16 | 54 (29.2) | 21 (30.0) |  |  |  |
| C*17 | 0 (0.0) | 2 (2.9) |  |  |  |
| C*18 | 0 (0.0) | 0 (0.0) |  |  |  |

The Table displays the univariate analysis for each HLA molecule. P-values are uncorrected

**Supplementary Table 4.** Unconditioned logistic regression model with the forms of the sequence 3-11 in leader peptide of the HLA-class I classical molecules found in our population.

| Peptide | P values | OR (95% CI) |
| --- | --- | --- |
| N1 | 0.29 |  |
| **N2** | **0.01** | **1.56 (1.11-2.21)** |
| N3 | 0.97 |  |
| N4 | 0.60 |  |
| N5 | 0.07 |  |
| N6 | 0.25 |  |
| N7 | 0.30 |  |
| N8 | 0.85 |  |
| N9 | 0.93 |  |
| N10 | 0.14 |  |

The Table displays the results of the multivariate analysis that includes all the nonapeptides in the model.

****Supplementary Table 5.** Distribution of the 2Met/Thr of HLA-B derivated-nonapeptide genotypes in BD patients and controls.**

| **2Met/Thr genotypes** | BD (%)  n= 435 | Controls (%)  n= 444 | p | OR (95% CI) |
| --- | --- | --- | --- | --- |
| MetMet | 16 (3.7) | 25 (5.6) |  |  |
| MetThr | 147 (33.8) | 179 (40.3) |  |  |
| ThrThr | **272 (62.5)** | **240 (54.1)** | **0.03** |  |
| Dominant Model* |  |  |  |  |
| Met | **163** | **204** | **0.01** | **0.71 (0.32-0.54)** |
| Thr | 419 | 419 |  |  |
| Recesive Model* |  |  |  |  |
| Met | 16 | 25 |  |  |
| Thr | **272** | **240** | **0.01** | **1.41 (1.08-1.86)** |
| Allelic Model | 2n= 870 | 2n= 888 |  |  |
| Met | 179 | 229 |  |  |
| Thr | **691** | **659** | **0.01** | **1.34 (1.07-1.68)** |
| B51-positive group | | | | |
|  | BD (%)  n= 185 | Controls (%)  n= 70 |  |  |
| MetMet | - | - |  |  |
| MetThr | 45 (24.3) | 17 (24.3) |  |  |
| ThrThr | 140 (75.7) | 53 (75.7) |  |  |
| B51-negative group | | | | |
|  | BD (%)  n= 250 | Controls (%)  n= 374 |  |  |
| MetMet | 16 (6.4) | 25 (6.7) |  |  |
| MetThr | 102 (40.8) | 162 (43.3) |  |  |
| ThrThr | 132 (52.8) | 187 (50.0) |  |  |

Univariate analysis of the **2Met/Thr of HLA-B** genotypes with different inheritance models. The Table also displays the frequency of the genotypes in B51 negative and positive individuals.

Dominant model AA+Aa versus aa; Recesive model AA versus Aa+aa

***By correcting on B51, the association of the 2Met, Thr HLA-B genotypes becomes non-significant P>0.05), whereas the association of B51 remains (P<10^-4^, OR=3.78, 95% CI 2.80-5.36)**

**Supplementary Table 6.** Frequency of **HLA-E genotypes, phenotypes and alleles in the B*51 positive and negative groups.**

| MODEL | HLA-E | B51-positive | | B51-negative | |
| --- | --- | --- | --- | --- | --- |
|  |  | BD (%)  n= 120 | Controls (%)  n= 36 | BD (%)  n= 153 | Controls (%)  n= 237 |
| Genotypic | 01:01/01:01 | 30 (25.0) | 7 (19.4) | 53 (34.6) | 65 (27.4) |
|  | 01:01/01:03 | 60 (50.0) | 16 (44.4) | 69 (45.1) | 123 (51.9) |
|  | 01:03/01:03 | 30 (25.0) | 13 (36.2) | 31 (20.3) | 49 (20.7) |
| Dominant | 01:01 | 90 (75.0) | 23 (63.9) | 122 (79.7) | 188 (79.3) |
|  | 01:03 | 90 (75.0) | 29 (80.6) | 100 (65.3) | 172 (72.6) |
| Recesive | 01:01 | 30 (25.0) | 7 (19.4) | 53 (34.6) | 65 (27.4) |
|  | 01:03 | 30 (25.0) | 13 (36.2) | 31 (20.3) | 49 (20.7) |
| Allelic | 01:01 | 120 (50.0) | 30 (41.7) | 175 (57.2) | 253 (53.4) |
|  | 01:03 | 120 (50.0) | 42 (58.3) | 131 (42.8) | 221 (46.6) |

HLA-E genotypes univariate analysis in B51 positive and negative patients. The Table also displays different inheritance models.

Dominant model AA+Aa versus aa. Recesive model AA versus Aa+aa

All the P values are >0.05.

**Supplementary Table 7.** **Frequency of the HLA-E genotypes according to the form of the nonapeptide 3-11 of the leader of the HLA class I classical molecules.**

| Peptides | HLA-E Genotypes | | | | |
| --- | --- | --- | --- | --- | --- |
|  |  | 01:01/01:01 | 01:01/01:03 | 01:03/01:03 | Total |
| N1 | BD (%) | 66 (31.9) | 103 (49.8) | 38 (18.4) | 207 |
|  | Controls (%) | 58 (26.2) | 112 (50.7) | 51 (23.1) | 221 |
| N2 | BD (%) | 64 (28.2) | 109 (48.0) | 54 (23.8) | 227 |
|  | Controls (%) | 48 (24.1) | 105 (52.8) | 46 (23.1) | 199 |
| N5 | BD (%) | 31 (31.3) | 51 (51.5) | 17 (17.2) | 99 |
|  | Controls (%) | 37 (29.8) | 66 (53.2) | 21 (16.9) | 124 |
| N6 | BD (%) | 46 (34.9) | 56 (42.4) | 30 (22.7) | 132 |
|  | Controls (%) | 33 (22.9) | 74 (51.4) | 37 (25.7) | 144 |
| N7 | BD (%) | 67 (29.8) | 108 (48.0) | 50 (22.2) | 225 |
|  | Controls (%) | 49 (24.7) | 103 (52.0) | 46 (23.2) | 198 |
| N8 | BD (%) | 70 (29.5) | 112 (47.3) | 55 (23.2) | 237 |
|  | Controls (%) | 63 (25.9) | 120 (49.4) | 60 (24.7) | 243 |
| N9 | BD (%) | 28 (27.2) | 49 (47.6) | 26 (25.2) | 103 |
|  | Controls (%) | 30 (30.3) | 48 (48.5) | 21 (21.2) | 99 |
| Total | BD (%) | 83 (30.4) | 129 (47.2) | 61 (22.3) | 273 |
|  | Controls (%) | 72 (26.4) | 139 (50.9) | 62 (22.7) | 273 |
